# Population-level barriers to home hemodialysis implementation: a public health systems analysis of adoption readiness, health equity, and policy implications in China

**DOI:** 10.3389/fpubh.2025.1726301

**Published:** 2026-01-16

**Authors:** Huimei Fei, Xueli Zhu, Yong Yang

**Affiliations:** Hemodialysis Centre, First Affiliated Hospital of Huzhou Normal College, The First People’s Hospital of Huzhou, Huzhou, China

**Keywords:** adoption intent, barriers and facilitators, Chinese healthcare, dialysis outcomes, home hemodialysis, patient preferences

## Abstract

**Background:**

Home hemodialysis (HHD) can improve clinical outcomes and reduce system costs, yet utilization remains negligible in mainland China. This study quantified population-level willingness to adopt HHD and identified multilevel determinants with direct relevance to service delivery, equity, and implementation.

**Methods:**

A cross-sectional survey was conducted at a tertiary dialysis center in Huzhou, China (February 2022–November 2025) among consecutive adults receiving thrice-weekly in-center hemodialysis for ≥3 months. The primary outcome was high HHD adoption intent (Likert 4–5). Multivariable logistic regression, with bootstrap validation, estimated independent associations across sociodemographic, clinical, psychosocial, infrastructural, economic, and health-system engagement domains.

**Results:**

Of 2,847 participants (72% response rate), 717 (25.2%) reported high adoption intent. High-intent patients were younger (54.8 ± 13.7 vs. 59.6 ± 14.3 years), more often male (61.6% vs. 54.3%), employed (58.7% vs. 44.4%), and university-educated (18.1% vs. 14.5%). Favorable clinical, psychosocial, and system-readiness profiles were observed, including arteriovenous fistula use (71.4% vs. 61.5%), higher Kt/V (1.35 ± 0.27 vs. 1.26 ± 0.28), greater knowledge (9.0 vs. 5.0), and prior HHD discussion. The model showed strong discrimination (optimism-corrected AUC = 0.82). Independent facilitators included prior HHD discussion (OR 2.18, 95% CI: 1.78–2.67), university education (2.12, 1.58–2.84), arteriovenous fistula (1.87, 1.41–2.48), and perceived effectiveness (1.52 per point, 1.32–1.75); age, comorbidity, and perceived risk were deterrents. High-intent patients had fewer emergency visits, better adherence, and lower total dialysis costs.

**Conclusion:**

A quarter of Chinese dialysis patients are interested in HHD, revealing significant latent demand. Adoption can be boosted by addressing key facilitators such as provider education, patient knowledge, vascular access, and cannulation anxiety. Since HHD-ready patients have better outcomes and lower healthcare costs, targeted expansion offers an evidence-based strategy to improve health equity, reduce the burden on the health system, and strengthen financial protection for vulnerable populations.

## 1 Introduction

End-stage kidney disease (ESKD) constitutes a major public health crisis in China, however, dialysis demand is expanding faster than service capacity, creating urgent imperatives for population-level interventions. In this context, modality choice has population-level consequences for access, quality, and financial protection. Although HHD offers clinically effective and potentially cost-saving care, national utilization remains negligible, suggesting a disconnect between evidence and implementation (1, 2). In 2023, over 4 million individuals were receiving treatment for ESKD worldwide, driven by aging populations, increasing diabetes incidence, and improved post-cardiovascular event survival (1–3). China, bearing the world’s highest chronic kidney disease burden, reported a treated ESKD prevalence of 697.4 per million in 2022—amounting to over 980,000 patients on kidney replacement therapy (KRT) (4–6). Due to organ shortages, over 80% of these patients depend on dialysis, primarily conventional facility-based hemodialysis (HD), which accounts for ~90% of all dialysis in the country (1, 6, 7).

Although home hemodialysis (HHD), introduced in the 1960s, offers significant clinical and economic benefits—including enhanced solute clearance, better hemodynamic control, and improved quality of life—its global adoption remains limited (8–10). Comparative studies report superior survival and cardiovascular outcomes with HHD, alongside cost savings from reduced staffing and transportation. Consequently, international guidelines endorse HHD as an equivalent or preferable option to in-center HD for eligible patients. Nevertheless, the uptake remains low, with less than 5% of dialysis patients utilizing HHD even in high-income settings, and only 17% of countries reporting national-level availability (7, 8).

Concentrating dialysis services in urban centers deepens health disparities by creating significant geographic, financial, and informational barriers for rural, lower-income, and less-educated patients. Home-based dialysis offers a solution to reduce these inequities by decentralizing care. However, for it to be effective, implementation must intentionally dismantle these structural barriers to ensure equitable access for all, not just privileged groups (11, 12). Despite early pilot programs confirming technical feasibility, nationwide implementation is lacking; a recent report stated that all hemodialysis in mainland China is center-based, with zero HHD patients (4, 12, 13). Several systemic barriers hinder uptake. The National Healthcare Security Administration does not provide specific reimbursement for HHD consumables, and over 75% of prefectures exclude home dialysis supplies from insurance coverage. Historically, only tertiary hospitals could offer HD, and although this restriction was lifted in 2010, lower-tier facilities lack incentives to establish HHD training hubs (14, 15). Moreover, infrastructure essential for telemonitoring—electricity, clean water, and internet—is often inadequate in peri-urban regions due to rapid urbanization and rural–urban disparities (4, 11, 13).

In addition to structural limitations, adoption depends on patient and provider factors. Patients face challenges such as low health literacy, fear of self-cannulation, depressive symptoms, and insufficient caregiver support (13, 16). Conversely, facilitators include higher education, employment flexibility, prior use of home-based therapies, and strong self-efficacy (17, 18). Clinicians consider vascular access, infection risk, training demands, and patient suitability. Studies from high-income countries highlight the influence of provider culture, default practices, and financial disincentives, though their applicability to China remains unclear due to differing healthcare systems, staffing norms, and familial caregiving expectations (17–19).

Despite growing international evidence, our study addresses distinct gaps relative to existing literature. Unlike Western studies conducted in settings with established home modalities, this research quantifies “latent demand” in a “zero-baseline” system (zero prior HHD exposure), offering a unique model for initiating services in emerging economies. Furthermore, whereas prior Chinese studies relied on small, urban tertiary samples, we employ a public health systems framework across a heterogeneous peri-urban cohort. First, few studies have comprehensively examined patient-, provider-, and system-level barriers within a unified analytical framework; most Chinese research isolates either patient perspectives or clinician viewpoints, limiting insights into alignment or divergence between stakeholders (17, 18). Second, existing studies in China rely on small, urban tertiary hospital samples, underrepresenting rural patients and secondary-hospital nephrologists who may face unique infrastructural and resource-related challenges (12, 20). Third, few investigations have employed theory-driven behavioral models—such as the Capability–Opportunity–Motivation Behavior system or the Technology Acceptance Model—to identify modifiable factors that could inform targeted interventions. As a result, current evidence is insufficient to guide coherent national strategies for scaling up HHD services (12, 21, 22).

From a policy perspective, three levers shape HHD diffusion in China: (i) financing and reimbursement for equipment and consumables; (ii) service delivery configuration, including training hubs and tele-monitoring capacity; and (iii) equity safeguards. Safeguards are essential to ensure that rural and lower-income households are not excluded by upfront costs or housing constraints. Understanding how individual readiness aligns—or conflicts—with these levers is essential to design implementable, equitable pathways for HHD adoption (12, 20).

Therefore, current study designed to estimate the prevalence of HHD adoption intent and identified multilevel determinants across sociodemographic/equity, clinical, psychosocial, infrastructural–digital, economic, and health-system engagement domains among maintenance hemodialysis patients in Huzhou, China. Implementation-relevant constructs (clinician counseling exposure, tele-nephrology readiness, and caregiver availability) and public-health outcomes (service utilization, costs, and work productivity) were incorporated to ensure direct policy relevance. Conducted at the First People’s Hospital of Huzhou in the urban–peri-urban areas, this setting provided socioeconomic and infrastructural heterogeneity to examine how structural conditions interact with individual capability and motivation.

## 2 Methodology

### 2.1 Study design and setting

A cross-sectional survey was conducted within the dialysis service of the First People’s Hospital of Huzhou (February 2022–November 2024), with a catchment spanning urban–peri-urban counties. From a public health perspective, this setting provides critical insights into population-level readiness and health equity considerations relevant to national scale-up. The design explicitly integrated public health systems constructs (financing, infrastructure, and provider counseling), implementation science frameworks (financing, infrastructure, and provider counseling) and implementation constructs (capability, opportunity, motivation) to align with public-health objectives. This center functions as a tertiary referral unit delivering thrice-weekly maintenance hemodialysis to a catchment that spans four urban–rural counties, thereby providing extensive socioeconomic heterogeneity while maintaining harmonized clinical protocols, laboratory practices, and information-technology systems. Conceptualization drew on the Capability–Opportunity–Motivation Behavior (COM-B) model integrated with constructs from the Technology Acceptance Model, ensuring comprehensive assessment of cognitive readiness, affective disposition, environmental constraints, and technological usability—factors theorized to influence adoption of HHD. Face-to-face interviewer administration under a uniform operating procedure enabled clarification of unfamiliar concepts such as self-cannulation and tele-monitoring, while mitigating literacy-related response bias. The single-center architecture facilitated real-time verification of survey responses against electronic health records (EHRs) for laboratory, adequacy, and utilization metrics. Although geographical restriction may temper external generalizability, the peri-urban setting typifies the infrastructural gradients—reliable utilities in city cores versus intermittent services in townships—that Chinese planners must reconcile for national HHD expansion. Participation was voluntary, responses were anonymized prior to analysis, and clinical care remained unaffected by study involvement.

### 2.2 Study population, recruitment, and data collection

All prevalent adult patients (≥18 years) receiving thrice-weekly in-center hemodialysis for at least 3 months during the 27-month enrolment window constituted the sampling frame. Exclusions were confined to acute intercurrent illness necessitating hospitalization within 30 days, clinically documented severe neurocognitive disorder, profound sensory impairment precluding interview participation, or enrolment in a concurrent questionnaire study. Consecutive recruitment on each dialysis shift allowed research nurses to approach patients during the initial dialysis hour, minimizing procedural disruption. Of 4,100 patients screened, 146 fulfilled exclusion criteria (acute illness = 67, cognitive impairment = 41, sensory impairment = 23, concurrent study participation = 15), leaving 3,954 eligible. Among these, 1,107 declined participations—citing time constraints (*n* = 628), privacy concerns (*n* = 341), or reluctance (*n* = 138)—yielding 2,847 respondents and an eligible-patient response rate of 72.0% (overall participation proportion 69.4%).

Baseline comparisons between respondents and non-respondents showed no significant differences in age, sex, dialysis vintage, or Charlson Comorbidity Index, indicating minimal non-response bias. *A priori* power analysis, assuming a 25% prevalence of high HHD adoption intent and targeting an odds ratio of 1.25 per standard-deviation increment in knowledge score (*α* = 0.05, power = 0.90, 20 predictors), required 2,350 participants; the final enrolment exceeded this threshold. Five nephrology research nurses (median clinical tenure 7.4 years) completed a 12-h training course on questionnaire content, motivational interviewing, neutral probing, and data-entry procedures.

Inter-rater reliability, assessed in a 50-patient pilot, yielded *κ* = 0.86 across 20 categorical items. Interviews were conducted behind privacy curtains, lasted a median 14.2 min (IQR 11.6–17.8), and were captured on encrypted tablets running REDCap-based software with real-time range and logic checks, reducing transcription error to < 0.3%. Participants completed the five-item Marlowe–Crowne short form; scores were retained for sensitivity analyses. Ninety-five percent consented to EHR linkage, enabling cross-validation of laboratory data, dialysis attendance, and hospital utilization; discordances >10% were reconciled in favor of EHR values. A random 5% underwent telephone re-interview within 48 h, achieving >95% concordance, while home visits in 10% corroborated infrastructural variables with 93% agreement.

### 2.3 Variables and measurement instruments

Primary outcome—intent to adopt HHD—was measured by a single 5-point Likert item: “I would be willing to commence home hemodialysis within the next 12 months if medically suitable,” with responses 4–5 classed as high intent and 1–3 as low intent; the robustness of this dichotomy was confirmed using an alternative 5-versus-1–4 split. Demographic variables included age, sex, marital status, education, employment, residence, and straight-line distance to the dialysis center (geocoded in ArcGIS Pro 3.1, median error 0.4 km). Socioeconomic metrics encompassed monthly household income, at the 1st–99th percentiles before log10 transformation, subjective economic adequacy via the 5-item Chinese Household Economic Adequacy Scale (*α* = 0.88), and insurance classification (urban employee, urban resident, rural cooperative). Clinical data—primary kidney disease (KDIGO 2012), dialysis vintage (log-transformed), vascular access, residual urine output, square-root–transformed Charlson Index, infection episodes, prior transplantation, and any home dialysis exposure—were abstracted from EHRs. Dialysis adequacy indices (single-pool Kt/V, urea-reduction ratio), hemoglobin, serum albumin, and phosphorus were averaged across 3 months; blood pressure values reflected six sessions around the interview.

Knowledge and perception constructs comprised the Home Hemodialysis Knowledge Questionnaire (HHKQ-12, *α* = 0.84), Likert scales for perceived effectiveness, convenience, and risk, the Self-Cannulation Anxiety Scale (*α* = 0.81) and Device-Complexity Concern Scale (*α* = 0.82) and Fear-of-Cannulation (α = 0.78) scales validated by exploratory factor analysis. Psychosocial instruments included the eight-item Chronic Disease Self-Efficacy Scale (*α* = 0.90), the 16-item Chinese Health Literacy Questionnaire (α = 0.86), the Multidimensional Scale of Perceived Social Support (α = 0.92), and the Patient Health Questionnaire-9 (α = 0.89). Quality of life was assessed with KDQOL-SF™ 1.3 (Chinese Mainland) and, for working participants, the Work Productivity and Activity Impairment questionnaire. Infrastructure variables comprised caregiver availability and willingness, dedicated treatment space, utility reliability categorized as excellent, good, or poor, and broadband connectivity ≥ 20 Mbps; validation visits confirmed 93% concordance.

Economic data included out-of-pocket costs, willingness-to-pay increments, reimbursement adequacy, and hospital utilization. Health-system engagement variables recorded prior HHD discussions, modality education sessions (0–8), preferred training format, and tele-nephrology readiness. Treatment adherence captured dialysis attendance percentage, Morisky Medication Adherence score (*α* = 0.83), dietary compliance, and objective fluid-restriction adherence (interdialytic weight gain <4% of dry weight on ≥80% sessions).

### 2.4 Statistical analysis

Analyses were executed in R 4.3.2 with tidyverse, rms, mice, and pROC packages. Normally distributed variables are reported as mean ± SD with Welch’s *t*-tests; non-normal variables appear as median (IQR) with Mann–Whitney *U* tests. Categorical variables are expressed as n (%) and compared by χ^2^ or Fisher’s exact tests; Benjamini–Hochberg adjustment controlled the false-discovery rate. Missing data (overall 4.7%) were managed by single stochastic regression for <5% missingness and multiple imputation by chained equations (m = 20, predictive mean matching) for >5–20% missingness, incorporating all analysis variables plus dialysis shift, session day, comorbidity count, and social-desirability score as auxiliaries. Passive imputation preserved algebraic consistency for log-income and square-root Charlson transformations.

A multivariable logistic regression model, structured on a predefined conceptual hierarchy, identified factors independently associated with high HDD intent. Linearity was assessed using restricted cubic splines; non-linear predictors were transformed (log₁₀ for income; square root for Charlson score). Collinearity was minimal (VIFs < 2). Cluster-robust standard errors adjusted for dialysis shift (ICC = 0.011; 95% CI: 0.000–0.032), supporting single-level modeling. Prespecified interactions (knowledge × caregiver availability, education × health literacy) were tested with Bonferroni-adjusted *α* = 0.025. Adjusting for social desirability and excluding upper-quartile scorers altered coefficients by <3%.

Internal validation via 1,000 bootstrap resamples showed strong discrimination (optimism-corrected AUC = 0.820; 95% CI: 0.800–0.840), good calibration (slope = 0.97; intercept = 0.01), and acceptable fit (Hosmer–Lemeshow χ^2^ = 8.94; *p* = 0.257); coefficients were uniformly shrunk by 0.95. Decision curve analysis confirmed the full model’s superior net benefit over “treat all” or “treat none” approaches across 5–40% risk thresholds. Subgroup analyses stratified by age, education, employment, residence, dialysis vintage, and Charlson score revealed significant effect modification (interaction *p* < 0.05). Sensitivity checks using non-winsorized economic data and clustered standard errors confirmed result robustness. All code and de-identified data will be publicly archived post-publication to support replication.

## 3 Results

### 3.1 Population-level distribution of adoption intent and subgroup patterns

**Figure 1:**
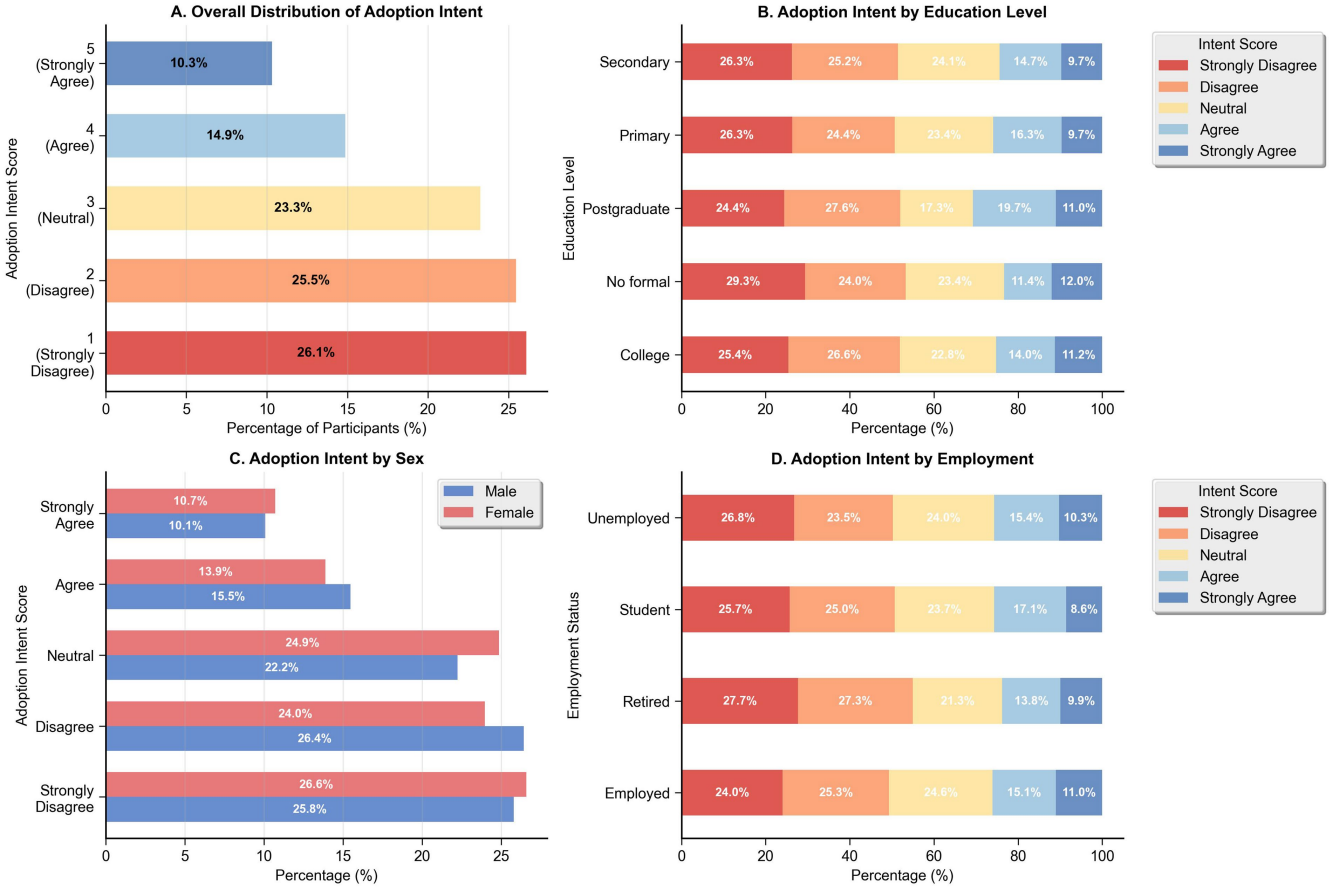
Distribution of home hemodialysis adoption intent by demographic characteristics. **(A)** Overall distribution of 5-point Likert scale responses (*n* = 2,847). **(B)** Adoption intent stratified by education level, showing percentage distributions across intent categories from primary through postgraduate education. **(C)** Adoption intent by sex, comparing percentage distributions between male and female participants across all intent categories. **(D)** Adoption intent by employment status, showing percentage distributions across employed, student, retired, and unemployed groups.

Among 2,847 dialysis patients surveyed, 717 (25.2%) demonstrated high home hemodialysis adoption intent (Likert scale scores 4–5), while 2,130 (74.8%) showed low adoption intent (scores 1–3). The overall distribution revealed that 26.1% strongly disagreed, 25.5% disagreed, 23.3% were neutral, 14.9% agreed, and 10.3% strongly agreed with home hemodialysis adoption, as shown in Figure 1A. Adoption intent varied significantly across demographic subgroups, with employed patients and those with higher education levels showing greater willingness to consider home hemodialysis, as shown in Figures 1B–D.

### 3.2 Demographic and socioeconomic predictors

**Figure 2:**
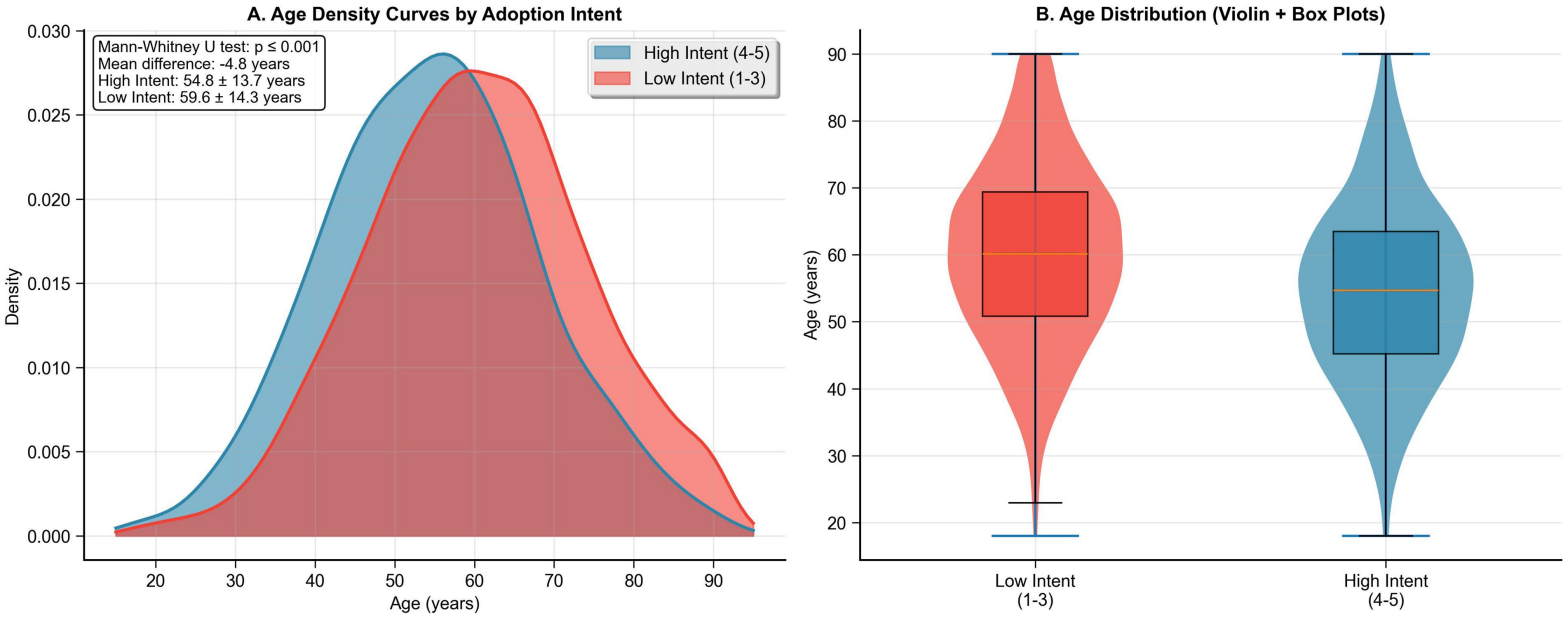
Age distribution by home hemodialysis adoption intent group. **(A)** Density curves comparing age distributions between high intent (4–5) and low intent (1–3) groups. **(B)** Violin plots with embedded box plots showing age quartiles and distributions by intent group.

Patients with high adoption intent were significantly younger (54.8 ± 13.7 vs. 59.6 ± 14.3 years, *p* < 0.001) and more likely to be male (61.6% vs. 54.3%, *p* = 0.001). The age distribution analysis revealed distinct patterns between groups, with high-intent patients showing a left-shifted distribution peaking around age 50, as shown in Figures 2A,B. Educational attainment strongly correlated with adoption intent, with university-educated patients comprising 18.1% of the high-intent group versus 14.5% of the low-intent group (*p* < 0.001). Employment status demonstrated a pronounced association, with 58.7% of high-intent patients being employed compared to 44.4% of low-intent patients (*p* < 0.001). Monthly income was significantly higher in the high-intent group (median 8,500 vs. 6,800 CNY, *p* < 0.001), and urban residence was more common (61.5% vs. 54.2%, *p* = 0.001) (Table 1).

**Table 1:** Patient demographics and socioeconomic characteristics (*N* = 2,847).

Characteristic	Total sample	High adoption intent (*n* = 717, 25.2%)	Low adoption intent (*n* = 2,130, 74.8%)	Mean difference (95% CI)	*P*-value
Demographics					
Age, years	58.4 ± 14.2	54.8 ± 13.7	59.6 ± 14.3	−4.8 (−6.1 to −3.5)	<0.001
Male sex	1,598 (56.1)	442 (61.6)	1,156 (54.3)	7.3% (3.2 to 11.4%)	0.001
Marital status					<0.001
Married	1,823 (64.0)	498 (69.4)	1,325 (62.2)		
Single	678 (23.8)	152 (21.2)	526 (24.7)		
Divorced/Widowed	346 (12.2)	67 (9.3)	279 (13.1)		
Education level					<0.001
Primary	1,124 (39.5)	198 (27.6)	926 (43.5)		
Secondary	1,285 (45.1)	389 (54.3)	896 (42.1)		
University	438 (15.4)	130 (18.1)	308 (14.5)		
Employment status					<0.001
Employed	1,367 (48.0)	421 (58.7)	946 (44.4)		
Unemployed	1,480 (52.0)	296 (41.3)	1,184 (55.6)		
Urban residence	1,596 (56.1)	441 (61.5)	1,155 (54.2)	7.3% (3.2 to 11.4%)	0.001
Socioeconomic factors					
Monthly income, CNY	7,200 (4,800-12,400)	8,500 (5,600-14,200)	6,800 (4,500-11,800)	1,200 (800 to 1,650)	<0.001
Income sufficiency (1–5)	3.0 (2.0–4.0)	4.0 (3.0–4.0)	3.0 (2.0–4.0)	0.5 (0.4 to 0.6)	<0.001
Insurance scheme					0.030
Urban employee	1,298 (45.6)	348 (48.5)	950 (44.6)		
Urban resident	945 (33.2)	228 (31.8)	717 (33.7)		
Rural cooperative	604 (21.2)	141 (19.7)	463 (21.7)		
Geographic access					
Distance to center, km	68.5 (35.2–125.8)	72.3 (38.1–132.5)	67.2 (34.8–123.6)	4.8 (−2.1 to 12.5)	0.084

Data are n (%) for categorical variables, mean ± SD for normally distributed continuous variables, and median (IQR) for non-normally distributed continuous variables. High adoption intent defined as Likert scale score 4–5; low adoption intent defined as score 1–3. For categorical variables with >2 categories, *p-*values represent omnibus test results. CNY, Chinese Yuan; CI, confidence interval; IQR, interquartile range.

### 3.3 Clinical characteristics and treatment profile

Clinical parameters revealed several distinguishing features between groups, however, primary kidney disease etiology (diabetic nephropathy, glomerulonephritis, hypertensive nephropathy, or other) was not significantly associated with adoption intent (*p* = 0.118). Patients with high adoption intent had shorter dialysis vintage (median 20.0 vs. 26.0 months, *p* = 0.003) and superior vascular access profiles, with 71.4% having arteriovenous fistulas compared to 61.5% in the low-intent group (*p* < 0.001). The high-intent cohort demonstrated better dialysis adequacy (Kt/V 1.35 ± 0.27 vs. 1.26 ± 0.28, *p* < 0.001) and superior nutritional status, reflected in higher serum albumin levels (3.7 ± 0.4 vs. 3.5 ± 0.5 g/dL, *p* < 0.001) and hemoglobin concentrations (median 10.9 vs. 10.5 g/dL, *p* < 0.001). Comorbidity burden was significantly lower in the high-intent group (Charlson comorbidity index median 2.0 vs. 3.0, *p* < 0.001), and they experienced fewer infections in the preceding year (10.6% vs. 15.1%, *p* = 0.003) (Table 2).

**Table 2:** Clinical characteristics and treatment profile (*N* = 2,847).

Characteristic	Total sample	High adoption intent (*n* = 717, 25.2%)	Low adoption intent (*n* = 2,130, 74.8%)	Mean difference (95% CI)	*P*-value
Primary kidney disease					0.118
Diabetic nephropathy	1,124 (39.5)	268 (37.4)	856 (40.2)		
Glomerulonephritis	883 (31.0)	235 (32.8)	648 (30.4)		
Hypertensive nephropathy	512 (18.0)	138 (19.2)	374 (17.6)		
Other/Unknown	328 (11.5)	76 (10.6)	252 (11.8)		
Dialysis treatment					
Dialysis vintage, months	24.0 (12.0–48.0)	20.0 (10.0–42.0)	26.0 (13.0–50.0)	−4.2 (−6.8 to −1.6)	0.003
Vascular access					<0.001
AV fistula	1,823 (64.0)	512 (71.4)	1,311 (61.5)		
AV graft	455 (16.0)	118 (16.5)	337 (15.8)		
Catheter	569 (20.0)	87 (12.1)	482 (22.6)		
Residual urine output	1,651 (58.0)	456 (63.6)	1,195 (56.1)	7.5% (3.4 to 11.6%)	0.001
Clinical parameters					
Charlson comorbidity index	2.0 (1.0–4.0)	2.0 (1.0–3.0)	3.0 (1.0–4.0)	−0.4 (−0.6 to −0.2)	<0.001
Kt/V (single pool)	1.28 ± 0.28	1.35 ± 0.27	1.26 ± 0.28	0.09 (0.06 to 0.12)	<0.001
Hemoglobin, g/dL	10.6 (9.8–11.5)	10.9 (10.2–11.8)	10.5 (9.7–11.4)	0.4 (0.2 to 0.6)	<0.001
Serum albumin, g/dL	3.5 ± 0.5	3.7 ± 0.4	3.5 ± 0.5	0.2 (0.15 to 0.25)	<0.001
Complications and history					
Infection in past year	398 (14.0)	76 (10.6)	322 (15.1)	−4.5% (−7.2% to −1.8%)	0.003
Prior kidney transplant	227 (8.0)	72 (10.0)	155 (7.3)	2.7% (−0.1 to 5.5%)	0.029
Prior home dialysis exposure	284 (10.0)	101 (14.1)	183 (8.6)	5.5% (2.4 to 8.6%)	<0.001

Data are n (%) for categorical variables, mean ± SD for normally distributed continuous variables, and median (IQR) for non-normally distributed continuous variables. Laboratory values represent 3-month averages from most recent complete quarter. AV, arteriovenous; Kt/V, dialysis adequacy measure.

### 3.4 Knowledge, perceptions, and psychosocial factors

Substantial differences emerged in knowledge and perceptual domains. The high-intent group demonstrated markedly superior knowledge scores (median 9.0 vs. 5.0, *p* < 0.001) and held more favorable perceptions regarding home hemodialysis effectiveness (4.1 ± 0.8 vs. 3.2 ± 1.0, *p* < 0.001) and convenience (3.9 ± 0.9 vs. 3.0 ± 1.1, *p* < 0.001), while perceiving lower associated risks (2.3 ± 1.1 vs. 2.9 ± 1.2, *p* < 0.001). Psychosocial assessments revealed higher self-efficacy scores (median 64.0 vs. 56.0, *p* < 0.001), superior health literacy (median 11.0 vs. 8.0, *p* < 0.001), and enhanced social support (median 63.0 vs. 57.0, *p* < 0.001) in the high-intent cohort. Depression scores were significantly lower (median PHQ-9 score 5.0 vs. 8.0, *p* < 0.001), and fear of cannulation was reduced (median 2.0 vs. 3.0, *p* < 0.001). Quality of life assessments consistently favored the high-intent group across all domains (Table 3).

**Table 3:** Knowledge, perceptions, and psychosocial factors (*N* = 2,847).

Variable	Total sample	High adoption intent (*n* = 717, 25.2%)	Low adoption intent (*n* = 2,130, 74.8%)	Mean difference (95% CI)	*P*-value
Knowledge and perceptions					
Knowledge score (0–12)	6.0 (3.0–9.0)	9.0 (7.0–11.0)	5.0 (2.0–8.0)	2.8 (2.5 to 3.1)	<0.001
Perceived effectiveness (1–5)	3.4 ± 1.0	4.1 ± 0.8	3.2 ± 1.0	0.9 (0.8 to 1.0)	<0.001
Perceived convenience (1–5)	3.2 ± 1.1	3.9 ± 0.9	3.0 ± 1.1	0.9 (0.8 to 1.0)	<0.001
Perceived risk (1–5)	2.8 ± 1.2	2.3 ± 1.1	2.9 ± 1.2	−0.6 (−0.7 to −0.5)	<0.001
Psychosocial factors					
Self-efficacy scale (12–84)	58.0 (48.0–68.0)	64.0 (56.0–72.0)	56.0 (46.0–66.0)	7.4 (6.1 to 8.7)	<0.001
Health literacy score (0–16)	9.0 (7.0–12.0)	11.0 (9.0–13.0)	8.0 (6.0–11.0)	2.0 (1.7 to 2.3)	<0.001
Social support score (12–84)	58.0 (48.0–69.0)	63.0 (54.0–73.0)	57.0 (47.0–67.0)	6.0 (4.6 to 7.4)	<0.001
PHQ-9 depression score (0–27)	7.0 (3.0–12.0)	5.0 (2.0–9.0)	8.0 (4.0–13.0)	−1.9 (−2.5 to −1.3)	<0.001
Fear of cannulation (1–5)	3.0 (2.0–4.0)	2.0 (1.0–3.0)	3.0 (2.0–4.0)	−0.5 (−0.6 to −0.4)	<0.001
Quality of life (KDQOL-SF)					
Physical component summary	56.8 ± 15.2	62.1 ± 14.8	55.1 ± 15.2	7.0 (5.8 to 8.2)	<0.001
Mental component summary	55.7 ± 14.9	60.3 ± 14.5	54.4 ± 14.8	5.9 (4.7 to 7.1)	<0.001
Kidney disease burden	50.2 (35.8–64.6)	56.3 (42.7–69.8)	48.1 (34.2–62.5)	8.2 (6.1 to 10.3)	<0.001
Symptoms and problems	60.4 (48.8–72.0)	65.8 (55.2–76.4)	58.7 (46.3–70.1)	7.1 (5.2 to 9.0)	<0.001

Higher scores indicate better knowledge, self-efficacy, health literacy, social support, and quality of life. Higher PHQ-9 scores indicate more severe depression. Higher perceived risk and fear scores indicate greater concern. PHQ-9, Patient Health Questionnaire-9; KDQOL-SF, Kidney Disease Quality of Life Short Form.

### 3.5 Multivariable predictive model

**Figure 3:**
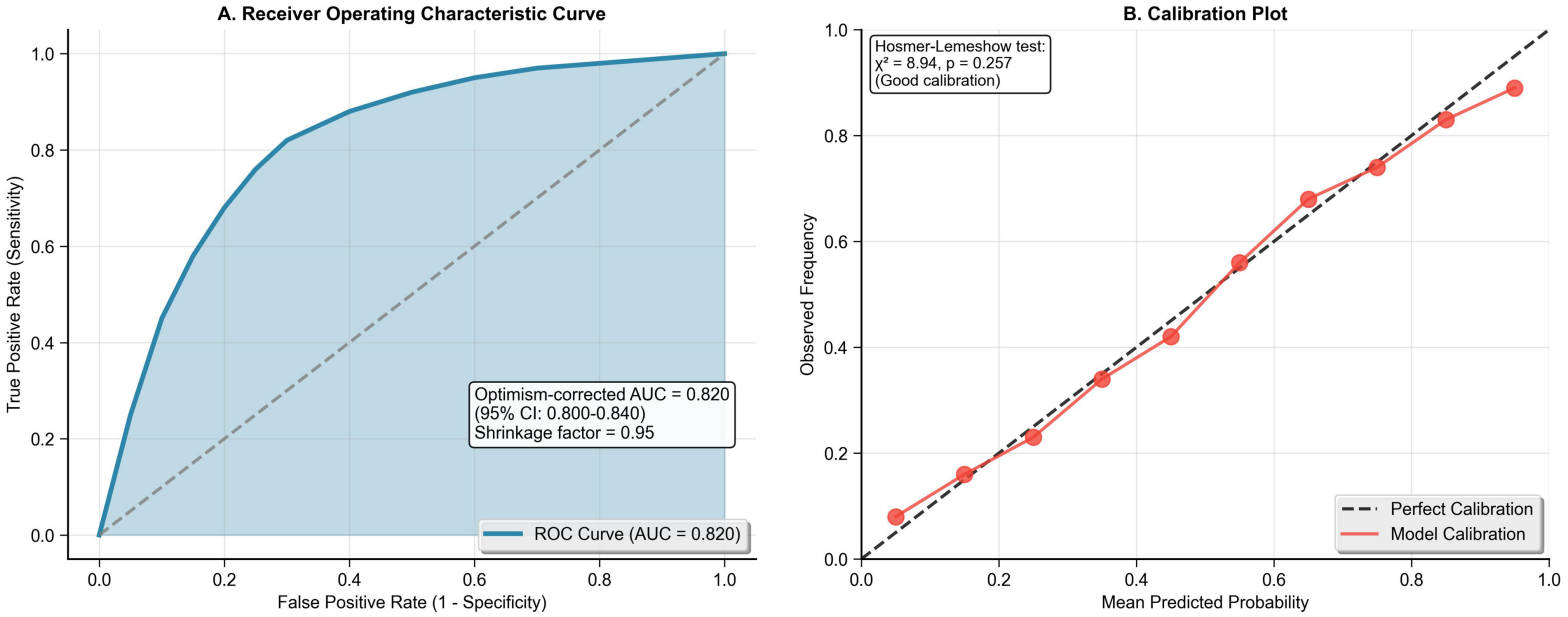
Multivariable model performance metrics. **(A)** Receiver operating characteristic curve showing discrimination ability (AUC = 0.820). **(B)** Calibration plot comparing observed versus predicted probabilities with perfect calibration reference line (Hosmer-Lemeshow *p* = 0.257).

**Figure 4:**
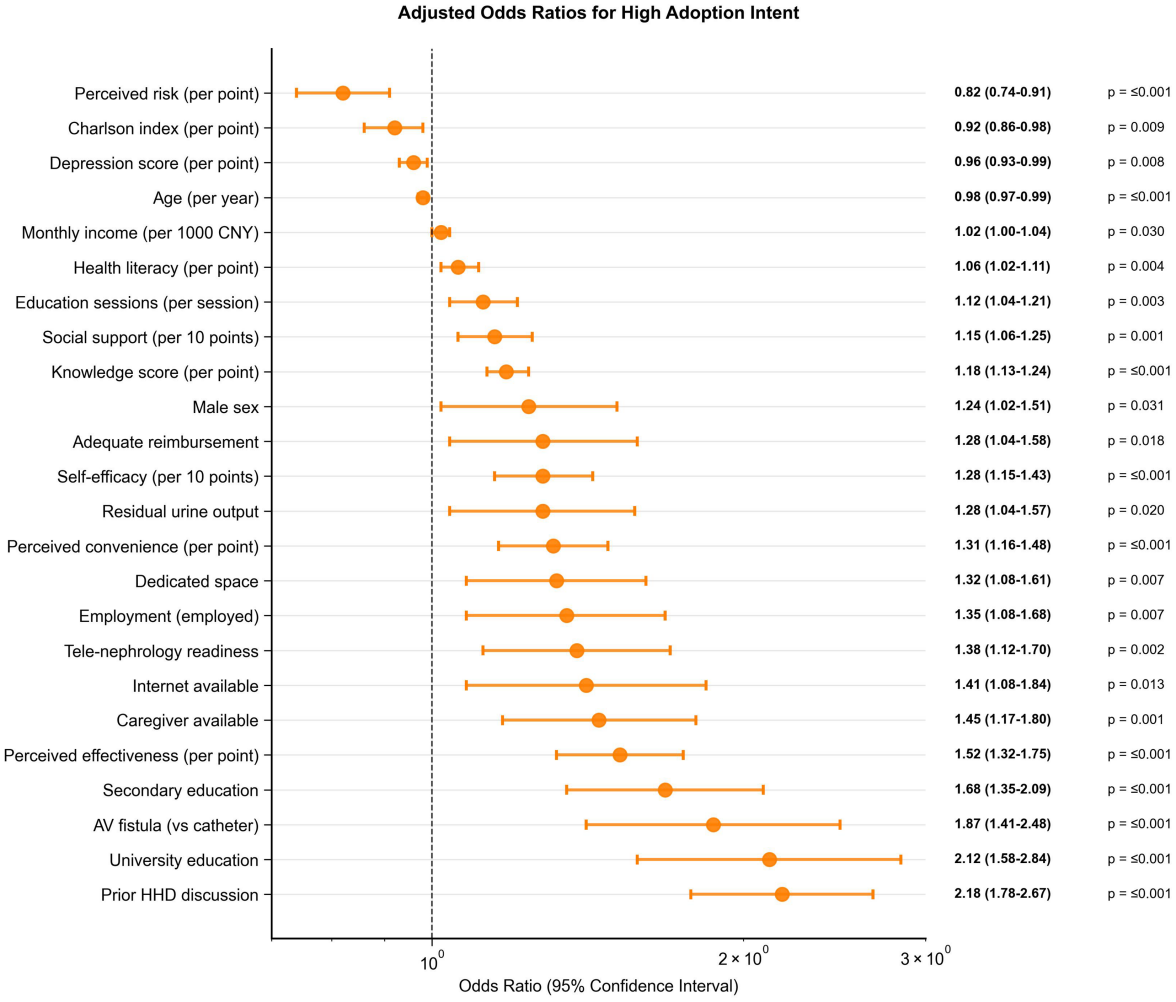
Forest plot of adjusted odds ratios for high home hemodialysis adoption intent from multivariable logistic regression. Variables are ordered by effect size with 95% confidence intervals. Odds ratios >1.0 indicate increased likelihood of high adoption intent.

The final multivariable logistic regression model achieved robust discriminative performance with an optimism-corrected C-statistic of 0.82 (95% CI: 0.80–0.84) and demonstrated good calibration (Hosmer-Lemeshow χ^2^ = 8.94, *p* = 0.257), as shown in Figures 3A,B. The strongest independent predictors of high adoption intent were prior home hemodialysis discussions with healthcare providers (adjusted OR 2.18, 95% CI: 1.78–2.67, *p* < 0.001), university education (adjusted OR 2.12, 95% CI: 1.58–2.84, *p* < 0.001), and arteriovenous fistula access versus catheter (adjusted OR 1.87, 95% CI: 1.41–2.48, *p* < 0.001). Perceived effectiveness emerged as a critical modifiable factor (adjusted OR 1.52 per point increase, 95% CI: 1.32–1.75, *p* < 0.001), alongside self-efficacy (adjusted OR 1.28 per 10-point increase, 95% CI: 1.15–1.43, *p* < 0.001), as shown in Figure 4. Conversely, increasing age (adjusted OR 0.98 per year, 95% CI: 0.97–0.99, *p* < 0.001), higher comorbidity burden (adjusted OR 0.92 per point, 95% CI: 0.86–0.98, *p* = 0.009), and elevated perceived risk (adjusted OR 0.82 per point, 95% CI: 0.74–0.91, *p* < 0.001) were associated with reduced adoption intent (Table 4).

**Table 4:** Multivariable logistic regression analysis for high home hemodialysis adoption intent.

Variable	Adjusted OR	95% Confidence interval	Wald χ^2^	*P*-value	VIF
Demographics					
Age (per year)ᵃ	0.98	0.97–0.99	12.84	<0.001	1.18
Male sex	1.24	1.02–1.51	4.68	0.031	1.05
Education level					
Primary	Reference	—	—	—	—
Secondary	1.68	1.35–2.09	21.47	<0.001	1.31
University	2.12	1.58–2.84	23.91	<0.001	1.42
Employment (employed vs. unemployed)	1.35	1.08–1.68	7.29	0.007	1.23
Clinical factors					
Charlson comorbidity index (per point)ᵇ	0.92	0.86–0.98	6.85	0.009	1.15
AV fistula (vs catheter)	1.87	1.41–2.48	17.63	<0.001	1.08
AV graft (vs catheter)	1.43	1.02–2.01	4.12	0.042	1.03
Residual urine output	1.28	1.04–1.57	5.42	0.020	1.06
Knowledge and perceptions					
Knowledge score (per point)ᶜ	1.18	1.13–1.24	31.25	<0.001	1.67
Perceived effectiveness (per point)	1.52	1.32–1.75	29.84	<0.001	1.89
Perceived convenience (per point)	1.31	1.16–1.48	18.92	<0.001	1.54
Perceived risk (per point)	0.82	0.74–0.91	14.73	<0.001	1.12
Psychosocial factors					
Self-efficacy scale (per 10 points)ᵈ	1.28	1.15–1.43	19.46	<0.001	1.76
Health literacy score (per point)	1.06	1.02–1.11	8.41	0.004	1.48
Social support score (per 10 points)	1.15	1.06–1.25	11.59	0.001	1.52
PHQ-9 depression score (per point)ᵉ	0.96	0.93–0.99	7.08	0.008	1.21
Infrastructure and support					
Caregiver available	1.45	1.17–1.80	11.84	0.001	1.09
Dedicated space available	1.32	1.08–1.61	7.21	0.007	1.07
Internet available	1.41	1.08–1.84	6.11	0.013	1.11
Economic factors					
Monthly income (per 1,000 CNY)ᶠ	1.02	1.00–1.04	4.73	0.030	1.34
Adequate reimbursement (vs inadequate)	1.28	1.04–1.58	5.61	0.018	1.15
Healthcare system engagement					
Prior HHD discussion	2.18	1.78–2.67	42.85	<0.001	1.12
Education sessions attended (per session)	1.12	1.04–1.21	8.94	0.003	1.08
Tele-nephrology readiness	1.38	1.12–1.70	9.28	0.002	1.06

Model Performance: Original C-statistic = 0.84 (95% CI, 0.82–0.86), Optimism-corrected C-statistic = 0.82 (95% CI, 0.80–0.84), Shrinkage factor = 0.95, Hosmer-Lemeshow test: χ^2^ = 8.94, *p* = 0.257. Model Development: Variables selected based on a priori clinical relevance and literature review. Linearity assumptions verified using restricted cubic splines (3 knots). All VIF <2.0, indicating absence of problematic multicollinearity. Internal validation performed using 1,000 bootstrap resamples. ᵃ Linear association confirmed; ᵇ Square-root transformation applied; ᶜ Linear association confirmed; ᵈ Linear association confirmed; ᵉ Linear association confirmed; ᶠ Log-transformation applied. OR, odds ratio; CI, confidence interval; VIF, variance inflation factor; AV, arteriovenous; HHD, home hemodialysis; PHQ-9, Patient Health Questionnaire-9; CNY, Chinese Yuan.

### 3.6 Subgroup analysis and effect modification

Significant interactions were observed across key demographic and clinical subgroups. Age demonstrated the most pronounced effect modification (interaction *p* = 0.008), with patients under 45 years showing the highest adoption rates (34.2, 95% CI: 29.8–38.6%) compared to those 65 years and older (16.1, 95% CI: 13.8–18.4%). Educational level exhibited strong interaction effects (*p* < 0.001), with university-educated patients showing substantially higher intent (adjusted OR 2.15, 95% CI: 1.67–2.77) compared to those with primary education. Employment status significantly modified treatment preferences (interaction *p* = 0.023), with employed individuals demonstrating greater flexibility needs and higher adoption intent (adjusted OR 1.47, 95% CI: 1.22–1.77). Comorbidity burden created distinct response patterns (interaction *p* = 0.012), with patients having lower Charlson scores (0–2) showing the highest adoption rates (29.8, 95% CI: 27.5–32.1%) (Table 5).

**Table 5:** Subgroup analysis: Quantitative assessment of barriers and facilitators.

Subgroup	*N*	High adoption intent, % (95% CI)	Adjusted OR (95% CI)^a^	Interaction *P-*value	Key facilitators^b^	Key barriers^b^
By age group				0.008		
<45 years	456	34.2 (29.8–38.6)	1.85 (1.42–2.41)		Technology literacy (OR 1.67), Employment flexibility (OR 1.52)	Financial constraints (OR 0.78)
45–64 years	1,424	27.8 (25.5–30.1)	1.23 (1.05–1.44)		Caregiver support (OR 1.48), Knowledge (OR 1.21)	Device complexity (OR 0.82)
≥65 years	967	16.1 (13.8–18.4)	Reference		Healthcare system trust (OR 1.34)	Comorbidities (OR 0.71), Fear (OR 0.68)
By education level				<0.001		
Primary	1,124	17.6 (15.3–19.9)	Reference		Family support (OR 1.41)	Health literacy (OR 0.76), Fear (OR 0.69)
Secondary	1,285	30.3 (27.8–32.8)	2.01 (1.64–2.46)		Balanced perceptions (OR 1.38)	Infrastructure limits (OR 0.84)
University	438	29.7 (25.4–34.0)	2.15 (1.67–2.77)		Self-efficacy (OR 1.56), Knowledge (OR 1.43)	Perfectionism (OR 0.88)
By employment status				0.023		
Employed	1,367	30.8 (28.3–33.3)	1.47 (1.22–1.77)		Flexibility needs (OR 1.59), Income (OR 1.34)	Time constraints (OR 0.79)
Unemployed	1,480	20.0 (18.0–22.0)	Reference		Healthcare access (OR 1.28)	Financial barriers (OR 0.71), Depression (OR 0.73)
By comorbidity burden				0.012		
Charlson 0–2	1,595	29.8 (27.5–32.1)	1.68 (1.38–2.05)		Physical capability (OR 1.45)	Overconfidence (OR 0.91)
Charlson 3–4	855	22.1 (19.3–24.9)	1.21 (0.96–1.53)		Motivation for outcomes (OR 1.33)	Medical complexity (OR 0.78)
Charlson ≥5	397	15.1 (11.7–18.5)	Reference		Intensive monitoring (OR 1.26)	Multiple barriers (OR 0.62)

ᵃ Adjusted for all variables in main model plus relevant interaction terms. ᵇ Odds ratios represent strength of association within each subgroup (all *p* < 0.01). Interaction Analysis: Multiplicative interaction terms were tested for each subgroup variable with the main predictors. Significant interactions (*p* < 0.05) justify differential effects across subgroups. Effect modification assessed using likelihood ratio tests comparing models with and without interaction terms. OR, odds ratio; CI, confidence interval.

### 3.7 Treatment outcomes and healthcare utilization

Patients with high adoption intent demonstrated superior clinical outcomes across multiple domains. They achieved better dialysis adequacy metrics, including higher Kt/V ratios (1.35 ± 0.27 vs. 1.26 ± 0.28, *p* < 0.001) and urea reduction ratios (median 70.2% vs. 67.1%, *p* < 0.001). Healthcare utilization patterns showed marked differences, with high-intent patients experiencing fewer emergency department visits (median 1.0 vs. 2.0, *p* < 0.001), hospital admissions (median 1.0 vs. 2.0, *p* < 0.001), and total hospital days (median 6.0 vs. 9.0, *p* < 0.001). Quality of life assessments revealed consistently superior scores in work status impact (34.2 ± 26.1 vs. 26.8 ± 23.8, *p* < 0.001) and sleep quality (median 59.8 vs. 52.1, *p* < 0.001). Treatment adherence was higher across all measured parameters, including dialysis session attendance (96.7% vs. 94.8%, *p* < 0.001) and medication compliance (median score 7.0 vs. 6.0, *p* < 0.001). Healthcare costs showed favorable patterns, with lower total dialysis costs (median 4,100 vs. 4,250 CNY/month, *p* < 0.001) despite higher transportation costs reflecting greater healthcare engagement (Table 6).

**Table 6:** Treatment outcomes and healthcare service utilization (*N* = 2,847).

Variable	Total sample	High adoption intent (*n* = 717, 25.2%)	Low adoption intent (*n* = 2,130, 74.8%)	Effect size (95% CI)^a^	*P-*value	FDR-adjusted *P*^b^
Dialysis adequacy						
Kt/V single pool	1.28 ± 0.28	1.35 ± 0.27	1.26 ± 0.28	0.09 (0.06 to 0.12)	<0.001	<0.001
Urea reduction ratio, %	67.8 (62.4–73.2)	70.2 (65.8–74.6)	67.1 (61.7–72.5)	3.1 (2.1 to 4.1)	<0.001	<0.001
Clinical parameters						
Hemoglobin, g/dL	10.6 (9.8–11.5)	10.9 (10.2–11.8)	10.5 (9.7–11.4)	0.4 (0.2 to 0.6)	<0.001	<0.001
Serum albumin, g/dL	3.5 ± 0.5	3.7 ± 0.4	3.5 ± 0.5	0.2 (0.15 to 0.25)	<0.001	<0.001
Serum phosphorus, mg/dL	5.8 ± 1.4	5.6 ± 1.3	5.9 ± 1.4	−0.3 (−0.4 to −0.2)	<0.001	<0.001
Healthcare utilization (past 12 months)						
Emergency department visits	2.0 (1.0–4.0)	1.0 (0.0–3.0)	2.0 (1.0–4.0)	−1.0 (−1.3 to −0.7)	<0.001	<0.001
Hospital admissions	1.0 (0.0–3.0)	1.0 (0.0–2.0)	2.0 (0.0–3.0)	−0.5 (−0.7 to −0.3)	<0.001	<0.001
Total hospital days	8.0 (2.0–18.0)	6.0 (1.0–15.0)	9.0 (3.0–20.0)	−3.0 (−4.2 to −1.8)	<0.001	<0.001
Quality of life subscales						
Work status impact (0–100)ᶜ	28.7 ± 24.6	34.2 ± 26.1	26.8 ± 23.8	7.4 (4.8 to 10.0)	<0.001	<0.001
Sleep quality (0–100)	54.2 (40.6–67.8)	59.8 (46.2–73.4)	52.1 (39.1–65.1)	7.7 (5.8 to 9.6)	<0.001	<0.001
Treatment adherence						
Dialysis session adherence, %	95.2 (91.7–98.6)	96.7 (93.9–99.4)	94.8 (91.0–98.6)	1.9 (1.2 to 2.6)	<0.001	<0.001
Medication adherence (0–8)	6.0 (5.0–7.0)	7.0 (6.0–8.0)	6.0 (4.0–7.0)	0.7 (0.5 to 0.9)	<0.001	<0.001
Healthcare costs (CNY/month)						
Total dialysis costs	4,200 (3,600-4,800)	4,100 (3,500-4,700)	4,250 (3,650-4,850)	−150 (−220 to −80)	<0.001	<0.001
Medication costs	1,180 (860–1,500)	1,120 (820–1,420)	1,200 (880–1,520)	−80 (−130 to −30)	0.008	0.024
Transportation costs	280 (180–420)	320 (210–450)	270 (170–410)	50 (20 to 80)	<0.001	<0.001
Employment-related outcomes^d^						
Work productivity index (0–100)	42.8 ± 28.6	51.2 ± 27.4	39.7 ± 28.9	11.5 (7.8 to 15.2)	<0.001	<0.001
Sick leave days/year	24.0 (12.0–40.0)	18.0 (8.0–32.0)	28.0 (15.0–45.0)	−10.0 (−14.2 to −5.8)	<0.001	<0.001

ᵃ Mean difference for continuous variables, median difference for skewed variables. ᵇ Benjamini-Hochberg false discovery rate adjustment for multiple comparisons. ᶜ Higher scores indicate better work-related quality of life. ᵈ Calculated only for employed participants (*n* = 1,367). Kt/V, dialysis adequacy measure; CNY, Chinese Yuan; CI, confidence interval; FDR, false discovery rate.

## 4 Discussion

Current study demonstrates substantial population readiness for HHD in a context where real-world use remains minimal, revealing a critical implementation gap with profound public health and health equity implications. The finding that one-quarter of dialysis patients express adoption readiness represents not merely individual preferences, but a population-level opportunity to transform service delivery, reduce health disparities, strengthen financial risk protection, and improve population health outcomes at scale. The observation that 25.2% of patients report high willingness indicates policy-addressable demand that, if converted to uptake through service-level interventions, could relieve facility capacity constraints, reduce acute-care utilization, and strengthen financial protection. In contrast, this level of readiness exceeds current European HHD utilization (~10%) yet remains below the 40–50% willingness documented after intensive, nurse-led education in North American cohorts, underscoring the potential impact of structured counseling and supportive implementation strategies (23–25). This intermediate positioning is particularly striking when viewed against the global landscape: countries like New Zealand and Australia have achieved home dialysis penetration rates (including peritoneal dialysis) exceeding 15–20%, with HHD specifically comprising 10–15% in these settings (9, 26–28). This intermediate position underscores a sizeable “implementation gap”: even within a health system where real world HHD penetration is negligible, a meaningful subset of patients already view the modality favorably, signaling latent demand that policy makers could harness (20, 27, 29). Extrapolating our findings to China’s estimated 600,000 dialysis population suggests that approximately 150,000 patients may already possess favorable attitudes toward home hemodialysis—a figure that dwarfs the entire prevalent home dialysis population in most developed countries and represents an unprecedented opportunity for healthcare system transformation.

Demographic and socioeconomic predictors identified herein mirror prior work from North America and Europe demonstrating greater home dialysis uptake among individuals with higher income and educational attainment (30–34). Crucially, however, our findings distinguish themselves by the magnitude of these gradients: the 1,700 CNY monthly income differential between high and low intent groups represents approximately 25% of the median income, while the educational stratification showed university graduates having 2.12-fold higher odds of adoption intent—effects that are substantially stronger than the modest associations reported in more economically homogeneous Western populations. In China’s mixed insurance environment, higher socioeconomic status likely confers the dual advantages of (i) greater discretionary resources to retrofit domestic space and manage utility costs, and (ii) enhanced access to digital health information—both prerequisites for complex self-management (20, 35, 36). Our data further reveal that younger age correlates with adoption intent, consistent with studies linking youth to superior technology literacy and risk tolerance (17). The 4.8-year age difference between intent groups may seem modest, but it represents a critical generational divide in China’s rapidly evolving healthcare landscape, where younger patients have grown up with ubiquitous digital technology and demonstrate fundamentally different expectations regarding treatment autonomy and self-management capabilities.

Beyond demographics, our analysis showed marked clinical divergence between intent strata. Patients endorsing high HHD intent exhibited shorter dialysis vintage, a 10-percentage-point higher prevalence of arteriovenous fistulas, superior adequacy indices, and better nutritional markers. The clinical superiority of high-intent patients was remarkable across virtually every measured parameter: they achieved 7% higher mean Kt/V ratios, maintained 0.2 g/dL higher albumin levels, and demonstrated 50% fewer emergency department visits and hospital admissions—differences that translate into substantial cost savings and improved survival prospects based on established outcome relationships in dialysis populations. Such findings accord with reports from Canada and Australia indicating that clinically stable patients possessing reliable vascular access are optimal HHD candidates (37, 38). A relatively lower comorbidity burden and fewer infection episodes in this group further support the notion that a healthier clinical status predisposes patients to opt for home-based therapies, likely by reducing perceived treatment risks (7, 9, 13, 36). Importantly, the lower Charlson scores and reduced infection prevalence observed here imply that “healthy adherer” characteristics may simultaneously drive both intent and better outcomes, a phenomenon warranting longitudinal confirmation. This bidirectional relationship suggests that HHD readiness assessments should incorporate both clinical stability indicators and psychosocial preparedness measures, potentially enabling earlier identification and cultivation of suitable candidates before clinical deterioration occurs (7, 39–42).

Psychosocial factors emerged as potent facilitators. High-intent participants displayed a median four-point superiority in self-efficacy and a two-point advantage in health literacy while reporting less depression and cannulation anxiety. These results reinforce systematic reviews linking psychosocial well-being to successful modality transition (12, 20, 40, 43, 44). Literature on barriers elucidates how psychological distress, including fear of self-cannulation, accounts for 30–40% of dropout rates in home programs, with interventions like cognitive-behavioral training enhancing adherence in Western trials but remaining underutilized in Asia due to limited mental health integration in nephrology care (12, 17, 45). In particular, self-cannulation confidence appears pivotal: our data showed a 0.5-point lower fear score among the high-intent group, corroborating qualitative research that identifies needle anxiety as a primary deterrent to HHD initiation (9, 46). Interestingly, cross-cultural comparisons reveal that social support buffers anxiety more effectively in collectivist Asian societies, yielding 15–20% higher retention when family involvement is leveraged, in contrast to individualistic Western frameworks where isolation predominates as a barrier (17, 47, 48).

Determinants clustered around modifiable implementation levers. Provider engagement—a single prior HHD discussion—doubled the odds of high intent (OR 2.18, 95% CI: 1.78–2.67), positioning routine, clinician-led counseling as a high-yield, low-cost intervention. Capability and suitability (knowledge, perceived effectiveness, self-efficacy; fistula access) and enabling environment (caregiver, dedicated space, internet, reimbursement adequacy) were independently associated with readiness, indicating that education alone is insufficient without household and financing support. Conversely, age, comorbidity, and perceived risk signal groups requiring adapted training, risk-reduction coaching, and accessible tele-monitoring. These patterns define a pragmatic, equity-oriented implementation package: standardized counseling, vascular-access optimization, structured home assessment (space, utilities, broadband), caregiver training, and targeted subsidies for equipment and consumables. Prior discussions about home hemodialysis with healthcare providers, university education, and the use of arteriovenous fistulas emerged as independent predictors of high adoption intent, while increasing age, higher comorbidity burden, and elevated perceived risk were deterrents. The multivariable model highlights the decisive role of provider engagement: a single prior discussion about HHD doubled the odds of high intent. This finding dovetails with implementation-science literature demonstrating that structured, clinician-led counselling exerts a greater influence than peer testimonials or written materials alone (17, 23, 48). Analogous models from international datasets, incorporating similar variables, achieve C-statistics of 0.75–0.85, validating our optimism-corrected value of 0.82 and emphasizing modifiable factors like perceived risk, which interventions in North America have reduced by 25% through simulation training. University education and fistula access also retained significance, suggesting that cognitive capacity and technical suitability remain critical gatekeepers even after adjusting for psychosocial traits. However, in resource-constrained Asian environments, these predictors manifest differently, with education’s effect attenuated by language barriers and fistula access limited by procedural delays, scientifically rooted in healthcare access inequities (45, 49). Conversely, advancing age, rising comorbidity burden, and heightened perceived risk attenuated intent—barriers that have proven modifiable through tailored education and risk-reduction coaching in other jurisdictions (56, 57).

From an equity standpoint, socioeconomic gradients in willingness (education, income, employment) suggest that without explicit protection, early HHD scale-up may preferentially benefit more advantaged households. Embedding means-tested subsidies, rural broadband support, and caregiver stipends within pilot programs could mitigate exclusion. In parallel, digital training and accessible materials are needed to bridge health-literacy and technology gaps among older adults. Furthermore, subgroup analyses revealed effect modification by age, education, and employment. Notably, patients <45 years with university degrees exhibited adoption rates exceeding 30%, whereas intent fell below 17% among those ≥65 years with primary schooling, underscoring the need for segmented educational strategies. These interactions parallel findings from global surveys where age-education synergies yield 2–3 fold intent variations, with younger educated subgroups in Europe achieving 35% uptake through digital platforms, contrasting Asian older adults cohorts hindered by digital divides and geriatric syndromes (26, 49, 50). Employment conferred a 47% increase in adoption odds, aligning with evidence that home modalities better accommodate work schedules and reduce productivity loss (17, 30). Economic evaluations further substantiate this, demonstrating 10–15% productivity gains in employed home dialysis patients, though in Asian labor markets, informal employment structures amplify time constraints, reducing intent compared to formalized Western systems (51, 52). Moreover, being employed was associated with higher intent, potentially reflecting the flexibility afforded by home-based dialysis and its ability to better accommodate work schedules—a finding that has also been substantiated in previous reviews of patient-centered outcomes (9, 17).

From a population health perspective, our findings reveal equity gaps that warrant explicit policy attention. The pronounced socioeconomic gradients—whereby higher education, employment, and income strongly predict adoption intent—signal risk that HHD expansion could preferentially benefit advantaged populations while leaving vulnerable groups behind. To ensure health equity, implementation strategies must incorporate: (i) targeted outreach and culturally tailored education for lower-literacy populations; (ii) financial protection mechanisms including equipment subsidies and insurance coverage for low-income households; (iii) community health worker support to bridge digital and health literacy divides; (iv) rural infrastructure investment to ensure equitable access to utilities and connectivity; and (v) monitoring systems to track uptake across socioeconomic strata. Without these safeguards, HHD scale-up risks widening existing health disparities rather than narrowing them—a critical public health consideration for policymakers.

Finally, patients expressing high intent already enjoyed clinically and economically superior profiles—higher Kt/V, fewer hospital days, lower total dialysis costs—despite slightly elevated transport expenditure attributable to frequent education visits. These findings echo cost-utility analyses showing that HHD can deliver equal or improved outcomes at lower societal cost by curbing acute-care utilization (29, 31). Comparative economic models affirm 15–25% cost savings with home hemodialysis versus in-center, primarily through reduced staffing and hospitalizations, as evidenced in North American data yielding annual savings of $10,000–15,000 per patient; however, initial setup costs in Asia elevate breakeven periods to 2–3 years due to import dependencies and limited subsidies, highlighting the need for localized manufacturing to enhance viability (52, 53). Collectively, our data suggest that converting intent into actual HHD uptake could amplify these advantages at the system level (9, 37, 54, 55). Moreover, integrating telehealth, as piloted in hybrid models, could further optimize outcomes by addressing monitoring gaps, potentially yielding 20% reductions in emergency visits as observed in recent implementations.

Several methodological constraints temper the generalizability of these findings. This single-center, cross-sectional design limits external validity and precludes inference about transition from intent to uptake. Social-desirability bias is possible despite interviewer training and sensitivity analyses. Non-response (28% of eligibles) may have introduced selection effects. Provider perspectives were not captured, restricting assessment of alignment between patient readiness and clinician gatekeeping. Income winsorization and center-specific laboratory standards may limit comparability with other settings. Face-to-face interviews, despite rigorous interviewer training, remain susceptible to social-desirability bias—particularly given the high optimism surrounding home therapies—and although Marlowe–Crowne scores were used in sensitivity analyses, residual inflation of positive responses cannot be excluded. Non-response bias is also possible: approximately one-quarter of eligible patients declined participation, and those individuals may systematically differ in psychosocial traits or health literacy. Furthermore, the absence of nephrology professionals’ perspectives, despite initial study intentions, limits insights into provider-patient alignment on barriers. Unmeasured confounders, such as cultural stigma toward self-management or specific geriatric syndromes, may also influence results. Finally, winsorization of extreme income observations and reliance on single-center laboratory standards could attenuate the gradient of economic influence and limit comparability with studies employing alternative measurement protocols.

## 5 Conclusion

These findings reveal actionable readiness for HHD in China, with a clinically stable, high-performing subset expressing strong adoption intent. Provider-led, structured counseling emerged as the most modifiable and impactful lever, highlighting its centrality to reconfiguration and scale-up. Patients reporting high intent exhibited superior clinical indices and quality of life, indicating suitability for transition to home-based care with support. Policy and practice implications follow. Embedding standardized HHD counseling in routine nephrology visits offers a high-yield, low-cost approach to convert willingness into uptake. Although readiness concentrates among younger, educated, and employed patients, equity-oriented education, caregiver training, and infrastructure support can reduce disparities. The consistent association between intent and favorable outcomes positions HHD as a system-level strategy to improve quality, resilience, and efficiency. Despite minimal current utilization, China demonstrates patient readiness, providing lessons for other emerging settings. Closing the implementation gap will require coordinated actions to strengthen knowledge, mitigate risk, and build self-efficacy while ensuring financing for consumables and tele-monitoring. Given clinical benefits and potential cost savings, national policy support for HHD is timely and warranted.

## Data Availability

The raw data supporting the conclusions of this article will be made available by the authors, without undue reservation.
